# Association of serum 25-OH-vitamin D level with FTO and IRX3 genes expression in obese and overweight boys with different FTO rs9930506 genotypes

**DOI:** 10.1186/s12967-021-03029-4

**Published:** 2021-08-16

**Authors:** Maryam Gholamalizadeh, Saeid Doaei, Zohreh Mokhtari, Vahideh Jalili, Fatemeh Bourbour, Saeed Omidi, Kamal Ebrahimi, Naser Kalantari, Sheyda Abdi, Ghasem Azizi Tabesh, Mohammad Naimi Joubani, Esmaeil Roohbakhsh, Seyed Alireza Mosavi Jarrahi

**Affiliations:** 1 Student Research Committee, Cancer Research Center, Shahid Beheshti University of Medical Sciences, Tehran, Iran; 2 Research center of Health and Enviroment, School of Health, Guilan university of Medical Sciences, rasht, Iran; 3 Department of Clinical Biochemistry, Faculty of Medicine, Shahid Beheshti University of Medical Sciences, Tehran, Iran; 4 Faculty of Medicine, Urmia University of Medical sciences, Urmia, Iran; 5 Department of Clinical Nutrition and Dietetics, Research Institute Shahid Beheshti University of Medical Science, Tehran, Iran; 6 Msc Student of Psychiatric Nursing, Qazvin University of Medical Sciences, Qazvin, Iran; 7 Department of community Nutrition and Dietetics, Research Institute Shahid Beheshti University of Medical Science, Tehran, Iran; 8 Department of Biochemistry and Dietetics, Faculty of Nutrition and Food Sciences, Tabriz University of Medical Sciences, Tabriz, Iran; 9 Department of Medical Genetics, School of Medicine, Shahid Beheshti University of Medical Sciences, Tehran, Iran; 10 School of Medicine, Shahid Beheshti University of Medical Sciences, Tehran, Iran

**Keywords:** Obesity, FTO gene, IRX3 gene, 25-OH-vitamin D

## Abstract

**Background:**

The roles of FTO gene and the level of serum 25-OH-vitamin D in obesity are frequently reported. This study aimed to investigate the interactions of serum 25-OH-vitamin D level, FTO and IRX3 genes expression, and FTO genotype in obese and overweight boys.

**Methods:**

This study was carried out on the 120 male adolescents with overweight in Tehran, Iran. Blood samples were collected from the participants in order to evaluate the serum level of 25-OH-vitamin D, the expression level of FTO and IRX3 genes, and FTO genotype for rs9930506 at baseline and after 18 weeks of the study.

**Results:**

In general, no significant association was found between serum 25-OH-vitamin D level and IRX3 and FTO genes expression. The results of linear regression on the relationship between 25-OH-vitamin D serum level and FTO and IRX3 genes expression based on FTO genotypes for rs9930506 indicated that in AA/AG genotype carriers, serum 25-OH-vitamin D level was positively associated with FTO gene expression (B = 0.07, p = 0.02) and inversely associated with IRX3 gene expression (B = − 0.07, p = 0.03). In GG carriers, serum 25-OH-vitamin D level was not associated with expression of IRX3 and FTO genes.

**Conclusion:**

There are significant interactions between 25-OH-vitamin D and the expression of FTO and IRX3 genes in the subset of obese patients with specific genotypes for FTO rs9930506. There was no association between serum 25-OH-vitamin D levels and the expression of FTO and IRX genes in individuals with a homozygous genotype for the risk allele of the FTO gene polymorphism.

## Introduction

Over 430 genes have been recognized to be associated with obesity and overweight [1, 2]. Among all of these, FTO (Fat mass and obesity-associated) gene with a high rate of genetic variations may has a key role in life-long obesity [3]. The FTO is the best-known obesity-related gene which can exert its effects through several mechanisms such as impacts on eating behaviors [4], energy homeostasis, and body fat storage [5]. Age-related effects of FTO gene on BMI have been reported in studies conducted on children, which was characterized by disturbed adiposity rebound and higher BMI in adulthood [6]. In addition, some studies found that the association between FTO gene and obesity is mediated by another genes. For example, the expression level of Iroquois-related Homeobox 3 (IRX3) gene in hypothalamus is associated with the amount of calorie intake and can be influenced by FTO gene polymorphisms [7].

On the other hand, recent studies have reported that the effect of genetic background on obesity and body composition can be affected by the state of intake of certain nutrients from the diet [8]. Some dietary components such as 25-OH-vitamin D may have some effects on the association between genes and obesity. It has been shown that serum 25-OH-vitamin D level is inversely related to obesity. The serum levels of 25-OH-vitamin D were found to be low among obese and overweight children and adolescents [9]. Other studies found that high fat mass percentages in girls and boys aged 6–12 were associated with 25-OH-vitamin D deficiency [10–12]. In a prospective study on children aged 5 to 12, serum 25-OH-vitamin D level was inversely related to the progression of obesity during the 29 months of follow-up [13]. Regarding the association between obesity-related genes and 25-OH-vitamin D, a recent study reported that 25-OH-vitamin D levels influence the effect size of FTO rs9939609 genotype on Roux-en-Y Gastric Bypass (RYGB) surgery-induced weight loss in obese patients [13].

Moreover, it’s reported that FTO genotype effects on obesity are more pronounced among people with insufficient 25-OH-vitamin D levels [14]. The association between serum 25-OH-vitamin D levels and FTO gene expression may be influenced by the FTO genotype. However, the association of 25-OH-vitamin D with FTO and IRX3 genes expression in people with different FTO genotype was not yet assessed. Therefore, this study aimed to investigate the interactions of serum 25-OH-vitamin D level, FTO and IRX3 genes expression, and FTO genotype in obese and overweight boys.

## Methods

### Study design

This study was carried out in February to July 2016 on the Iranian male adolescents aged 12 to 16 years. Two high schools were selected by random cluster sampling method from district 5 of Tehran, Iran. The inclusion criteria for schools were that they had no history of continuous education and training in the field of nutrition and physical activity. Also, they were similar in terms of socioeconomic status. The participants’ height was measured with tape meter, and their weight, BMI, body fat percentage (BF%), and skeletal muscle percentage (SM%) was measured using a bio impedance analyzer scale (Omron BF 510; Omron Corp, Kyoto, Japan). Five ml of Blood samples were collected from the participants in order to evaluate the serum level of 25-OH-vitamin D, the expression levels of FTO and IRX3 genes, and FTO genotype at the beginning and after 18 weeks of the study. All blood samples were collected at the same hours of daytime (between 11 am and 2 pm).

A total of 120 students participated in the study. Of these, 8 students refused to continue their cooperation because of different reasons (such as parents’ dissatisfaction with blood sampling, interference with curricula, and fear of blood sampling). Finally, 112 students were entered in blood sampling section. The inclusion criteria were age between 12 to 16, be obese or have overweight based on BMI for age and sex, the consent to participation of the students and their parents, lack of illnesses affecting weight, non-use of drugs affecting weight, and not having diet and exercise programs interfering with weight.

### Serum 25-OH-vitamin D measurements

A direct competitive enzyme-linked immunosorbent assay (ELISA) method and 25-OH-vitamin D VIDAS Kit (Marcy-l'Étoile, bioMérieux, France) were used for measuring serum 25-OH-25-OH-vitamin D level. The VIDAS 25-OH-vitamin D total assay is considered as a suitable measuring method for vitamins D2 and D3 serum levels with a high accuracy. Correlation between the results from VIDAS Kit with the reference methods of chromatography and volume spectrometry was r = 0.93 which indicated the high efficiency of this method.

#### Lifestyle assessment

A validated physical activity tracker (Xiaomi model mi band, China) was used for 3 consecutive days to evaluate the level of physical activity of the participants [15]. This device measures the level of physical activity in different conditions, such as walking and exercise in horizontals, vertical, and diagonal axes. It reports the number of steps, the distance in meters, the energy consumption in kilo-calories and the duration of the activity in minutes. Also, a valid semi-quantitative food frequency questionnaire (FFQ) was used to estimate the amount of calorie intake [16].

#### Measurement of gene expression

In order to evaluate the expression of FTO gene, blood samples were centrifuged at 2500 × g for 15 minutes and buffy coats were then separated. The RNA was extracted using RNA extraction kit (Gene All Company, South Korea) according to the manufacturer's instruction. The complementary DNA (cDNA) was then synthesized using cDNA synthesis kit (Gene All Company, South Korea) and were kept in 80 °C until real-time PCR was performed. The GeneRunner and Allele ID 4.0 (www.ZeroG.com) software products were used to design the primers. The real-time PCR method (Bio-Rad Co.) using SYBR Green was applied to determine the expression level of FTO and IRX3 genes using prepared solution including 12.5 μL of SYBR Green PCR Master Mix, 0.8 μl of forwarding primer (300 nM), 0.8 μl of reverse primer (300 nM), and 8.9 μl of water. Changes in the expression level of FTO and IRX3 genes were assessed using the HPRT gene as reference gene and presented by threshold cycle (CT) which indirectly indicates the concentration of the existing cDNA strands. In order to determine the FTO gene expression quantity, the Rest (v2.0.7; 2009) software and the ΔΔCT method were used.

#### Genotyping

The DNA extraction kit (Gene All Company, South Korea) was used to extract and purify DNA samples. The NanoDrop device (Thermo Scientific, Wilmington, DE, USA) was used to quantify DNA concentration. The optical density (OD) of the samples was measured at a wavelength of 260–280 nm. The quality of the extracted DNA was checked by agarose gel electrophoresis. In brief, genomic DNA was amplified by PCR using the Taq DNA Pol 2X Master Mix Red (Cat. No. A180301; Ampliqon, Denmark) and the PCR products were sequenced by GeneAll. The rs9930506 SNP in FTO gene was genotyped in all of the smaples and the quality and average length of the sequences were assessed using the Chromas software (version 2.33, https://www.Technelysium.com.au/chromas.html).

### Statistical analysis

Normal distribution of the dependent variables was investigated using Shapiro-Wilk test and Skewness and Kurtosis indices. Independent t-test method was used to compare the obese and overweight adolescent boys. Linear regression method was used to test the linear association of the level of serum 25-OH-vitamin D with FTO and IRX3 genes expression level after adjusting for confounders including age, calorie intake, BMI, fat mass, skeletal mass, and physical activity. SPSS software version 23 was used to analyze the data and the significance level for all analyses was considered as 0.05.

## Results

At baseline, the obese participants had higher body fat% (24.12 vs 31.17%, P = 0.01) and lower skeletal muscle% (36.47 vs 33.67%, P = 0.01) compared to the overweight participants (Table 1). No significant difference was found between two groups in terms of calorie intake, physical activity, and FTO and IRX3 genes expression level.

**Table 1:** Characteristics of study participants in obese and overweight adolescent boys at baseline

	Overweight (n = 52)	Obese (n = 50)	P
Age (year)	13.89 ± 0.914	13.90 ± 0.918	0.67
Height	168 ± 8.76	171 ± 8.95	0.13
Weight	68 ± 8.67	83 ± 10.02	0.01
BMI (Kg/m^2^)	23.57 ± 1.62	29.16 ± 2.94	0.01
FM (%)	24.12 ± 5.66	31.17 ± 5.02	0.01
SM (%)	36.47 ± 2.39	33.67 ± 2.09	0.01
PA (MET-minute per week)	1744 ± 2355.46	1894 ± 2598.27	0.31
Calorie intake (kcal/d)	2168 ± 772.35	2192 ± 1134.02	0.92
FTO gene expression	1.57 ± 1.93	0.68 ± 1.6	0.5
IRX3 gene expression	− 4.38 ± 2.17	− 4.17 ± 1.47	0.38

*BMI* body mass index, *FM* fat mass, *SM* skeletal mass, *PA* physical activity

Carriers of different FTO genotypes were compared regarding to body composition and lifestyle (Table 2). The participants with GG genotype of FTO rs9930506 had higher BMI, FM% and SM% compared to the AA/AG genotype carriers.

**Table 2:** Comparison of body composition and lifestyle between the participants with different FTO genotypes

	AA/AG (n = 196)	GG (n = 37)	P
Age(year)	13 ± 1.01	14.22 ± 3.11	0.48
Height	169.6 ± 8.85	169.2 ± 9.22	0.83
Weight	74.51 ± 13.27	79.67 ± 13.09	0.07
PA (MET-minute per week)	1123 (2146)	1189 (1334)	0.8
BMI (Kg/m^2^)	21.8 (4.65)	25.6 (4.97)	0.01
FM (%)	18.59 (8.33)	26 (8.6)	0.01
SM (%)	38.23 (3.57)	35.72 (3.60)	0.01
Calorie intake	2485 (881)	2420 (730)	0.6

*BMI* body mass index, *FM* fat mass, *SM* skeletal mass, *PA* physical activity

Linear regression identified no significant association between serum 25-OH-vitamin D level and FTO and IRX3 genes expression (β = 0.03, P = 0.25 and β = − 0.16, P = 0.34, respectively) (Table 3).

**Table 3:** Linear regression of the association between the level of 25-OH-vitamin D and FTO and IRX3 genes expression

	FTO gene		IRX3 gene	
	B	P	B	P
Model 1	0.18	0.6	− 0.03	0.93
Model 2	0.03	0.25	− 0.16	0.34

Model 1: crude, Model 2: Adjusted for age, calorie intake, body mass index, fat mass, skeletal mass, and physical activity

The results of linear regression of the relationship between 25-OH-vitamin D serum level and FTO and IRX3 genes expression based on FTO genotypes for rs9930506 indicated that in AA/AG genotype carriers, serum 25-OH-vitamin D level was positively associated with FTO gene expression (β = 0.07, p = 0.02) and inversely associated with IRX3 gene expression (β = − 0.07, p = 0.03) (Table 4). In GG carriers, serum 25-OH-vitamin D level was not associated with expression level of IRX3 and FTO genes (P = 0.31, P = 0.23, respectively).

**Table 4:** Linear regression of the association between the serum level of 25-OH-vitamin D and FTO and IRX3 genes expression based on FTO genotypes for rs9930506

	AA/AG				GG			
	FTO		IRX3		FTO		IRX3	
	B	P	B	P	B	P	B	P
Model 1	0.06	0.02	− 0.06	0.03	0.04	0.018	− 0.03	0.35
Model 2	0.07	0.02	− 0.05	0.03	0.04	0.23	− 0.08	0.31

Model 1: crude, Model 2: Adjusted for age, calorie intake, body mass index, fat mass, skeletal mass, and physical activity

## Discussion

In this study, no association was found between serum level of 25-OH-vitamin D and expression of IRX3 and FTO genes in overweight and obese participants. However, in subgroup analysis based on the FTO genotype for rs9930506 polymorphism, serum 25-OH-vitamin D concentration was positively associated with FTO gene expression (p=0.02) and inversely associated with IRX3 gene expression in AA/AG genotype carriers. In other hand, no significant association was found between 25-OH-vitamin D serum level and expression of IRX3 and FTO genes in people who were homozygous for the FTO G-variant. Up to now, to the best of our knowledge, no studies have examined the association between 25-OH-vitamin D and the expression of FTO and IRX3 genes in obese and overweight children. Moreover, this is the first study reporting a significant association between 25-OH-vitamin D and the expression of FTO and IRX3 genes in people with specific genotypes for FTO rs9930506.

A significant association between FTO gene polymorphism and the risk of obesity was elucidated in various populations, suggesting the SNPs of the first intron of the FTO gene are related with adiposity [17]. Several investigations have demonstrated an association between the FTO gene and obesity, implying it as a target gene for obesity investigations [18]. The high expression of FTO gene in the liver, hypothalamus, and visceral fat have been identified, which contributes to appetite, food intake, and inflammatory states [19, 20]. In recent studies, several variants of the FTO gene including rs9930506, rs178117449, rs7202116, rs3751812, rs1421085, and rs9939609 have been related with obesity [21]. FTO rs9930506 polymorphism is demonstrated to have a strong effect on body composition, body weight, and, BMI [20]. In addition, the upregulation the FTO gene was reported to be associated with the higher food intake [21], higher appetite, and higher desire for high- calorie foods [22]. Furthermore, in carriers of the FTO obesity risk allele, lipolysis of fat cell is decreased, highlighting the possible role of FTO gene in fat metabolism [23]. Merra et al. reported that FTO rs9939609 polymorphism is related to BMI and body composition and specifically with fat mass percentage [24]. Interestingly, Speakman indicated that FTO gene polymorphisms mediate their obesity effects via nearby genes such as RPGRIP1L and IRX3 [25].

In this study, we identified that 25-OH-vitamin D concentration was inversely associated with IRX3 gene expression in FTO rs9930506 AA/AG genotype carriers. The main molecular mechanism of FTO gene on body weight, BMI, and obesity has not yet been elucidated. However, it has been reported that FTO genotype had a strong association with obesity via altering the IRX3 gene expression level [26]. Recent investigations proposed that IRX3 and FTO genes are functionally connected and many of the FTO gene polymorphisms effects are applied through regulation of IRX3 gene expression level [27]. Accordingly, FTO may be a bystander gene anchoring highly conserved non-coding elements (HCNEs) that induce IRX3 expression [28]. Furthermore, IRX3 expression in hypothalamus could be related with calorie intake. Smemo et al. determined that the IRX3 gene expression level is regulated by a region in the intron 1 of the FTO gene and genetic variations in this specific intronic region can affect the IRX3 expression level [28]. In a similar study, it has been reported that adipocyte- specific expression of IRX3 was up-regulated with the existence of the FTO risk allele in lean adolescents, while it was unaffected by risk haplotypes in obese children [26].

In this study, a significant interaction between 25-OH-vitamin D status and FTO gene expression in AA/AG genotype carriers was identified. In line with our results, Abboud et al. evaluated children with variants in the FTO gene and demonstrated that FTO genotype rs9939609 was related with a considerable weight gain in children with 25-OH-vitamin D deficient (< 75 nmol/L) [29]. However, no significant genetic effects were reported in children who had sufficient serum level of 25-OH-vitamin D [29]. In another study, Lourenço et al. investigated the effect of FTO rs9939609 on BMI-for-age Z score and BMI variations during childhood in a longitudinal and population-based study and assessed whether these effects were changed by 25-OH-vitamin D status. It was identified that 25-OH-vitamin D status significantly altered the effects of FTO genotype on weight gain (P = 0.02) [10]. Moreover, the risk allele of rs9939609 was related with a 0.05 Z/year enhance in BMI for- age Z score in 25-OH-vitamin D–deficient children (P = 0.003), whereas no statistically significant genetic effects were identified among children with sufficient 25-OH-vitamin D levels [10]. For evaluating whether the magnitude of RYGB surgery-induced weight loss after two years is related with pre-surgery 25-OH-vitamin D status (< 50 nmol/L equals deficiency) and FTO genotype, Bandstein et al. examined the role of 25-OH-vitamin D and FTO in the weight loss effect of RYGB surgery in obese patients [30]. It was revealed that pre-surgery 25-OH-vitamin D status affects the size of FTO rs9939609 genotype influences on RYGB surgery-related weight loss in obese subjects [30].

The exact mechanisms underlying the interactions between FTO gene and serum 25-OH-vitamin D level is not yet clear. The higher expression level of FTO gene in the human brain has been identified, and there is evidence of relation between reduced cerebrocortical insulin sensitivity and FTO risk allele [31]. Previous studies have determined an important role for insulin in the regulation of body weight and energy homeostasis in the central nervous system (CNS) [32]. FTO genetic polymorphism may influence dietary intake, appetite, food choice, and weight gain from an early age through effects on insulin secretion [33]. Serum 25-OH-vitamin D level was also reported to play a critical role in appropriate insulin secretion and activity among adolescents [34]. Furthermore, a randomized controlled trial (RCT) in obese young adults demonstrated that supplementation with 25-OH-vitamin D could promote markers of insulin sensitivity and resistance [35]. A potential mechanism for the interaction between 25-OH-vitamin D status and FTO genotype might comprise insulin action in the CNS.

However, this study had some limitations. First, this study cannot prove a cause-and-effect relationship. Second, this study was performed only in obese adolescent boys and is not generalizable to other ages, females, and normal weight and underweight individuals. Third, the sample size was small, which may negatively affect the strength of the results. Future studies with larger sample sizes and including both sexes are needed to confirm the findings of this study on the relationship between serum levels of 25-OH-vitamin D with the expression of genes associated with obesity and the effect of FTO polymorphisms on this association.

## Conclusion

Taken together, there are significant interactions between 25-OH-vitamin D and the expression of FTO and IRX3 genes in the subset of obese patients that carriers’ specific genotypes for FTO rs9930506. The underlying pathways involved in the interaction between 25-OH-vitamin D status and FTO and IRX3 expression still need to be elucidated. Advances in nutritional genomics, the identification of interactions between genes and diet, and identifying the genotype of obesity-related genes can help clinicians and nutritionists in managing the obese individuals and recommend specific strategies to prevent obesity.

## Data Availability

Not applicable
